# UNCOMMON JEJUNOJEJUNOSTOMY PERFORATION: A CASE STUDY ON COMPLICATIONS FOLLOWING ROUX-EN-Y GASTRIC BYPASS

**DOI:** 10.51894/001c.123127

**Published:** 2024-08-31

**Authors:** Stephanie Agudelo

**Affiliations:** 1 College of Osteopathic Medicine Michigan State University

## 97

### INTRODUCTION

Roux-en-Y gastric bypass (RYGB) is the gold standard for weight loss surgeries, offering increased long-term weight loss and fewer complications compared to other bariatric procedures. Gastrojejunostomy (GJ) and jejunojejunostomy (JJ) creation provides restrictive and malabsorptive properties, suitable for patients with failed conservative measures, insulin resistance, severe GERD, and other GI diseases. RYGB complications include malabsorption issues like dumping syndrome and metabolic dysregulation. This case report investigates an uncommon perforation at the JJ site following routine RYGB, aiming to shed light on this less common complication.

### CASE DESCRIPTION

A 54-year-old woman presented to the emergency department with a one-day history of severe abdominal pain. She indicates a history of RYGB surgery about six weeks prior. Abdominal computed tomography (CT) revealed GI tract perforation with secondary peritonitis and a subsequent diagnostic laparoscopic surgery was performed. Although rare (1%), assessing an anastomotic leak risk is crucial during RYGB, considering risk factors like oxygen dependency, hypoalbuminemia, sleep apnea, hypertension, and diabetes. The patient, with medically controlled diabetes and hypertension, showed a 1cm hole at the JJ anastomosis during diagnostic laparoscopy. A repair with 3-0 absorbable sutures and a vascularized pedicle flap using the omentum was performed. A G-tube was placed in the remnant stomach due to an unexplained JJ anastomotic perforation.

### DISCUSSION

Early identification of postoperative anastomotic leaks is vital for optimal management. The patient, promptly assessed in the emergency department, was correctly diagnosed with a GI bleed. While the patient was cleared for a solid food diet the day prior, the role of solid foods in the JJ anastomotic leak requires further investigation, necessitating more evidence to determine specific risk factors for this postoperative complication.

